# FIB-4 and APRI scores for progressive liver fibrosis diagnosis in children with biliary atresia

**DOI:** 10.3389/fped.2023.1286400

**Published:** 2024-01-05

**Authors:** Hongyu Lyu, Yongqin Ye, Bin Wang

**Affiliations:** ^1^ Department of General Surgery, Shenzhen Children’s Hospital of China Medical University, Shenzhen, China; ^2^ Faculty of Medicine, Macau University of Science and Technology, Macau, Macau SAR, China

**Keywords:** predictive value of tests, liver fibrosis, children, biliary atresia, aspartate aminotransferase, platelet

## Abstract

**Introduction:**

Finding non-invasive methods to predict the degree of liver fibrosis is very important in managing children with biliary atresia. Therefore, we explored the predictive value of APRI, FIB-4, and serological markers for liver fibrosis in children with biliary atresia.

**Methods:**

This study retrospectively reviewed data from children diagnosed with BA between March and December 2022. Liver tissue pathology specimens were obtained during surgery. The serum markers were measured within 2 days before the Kasai procedure or liver transplantation. The aspartate aminotransferase-to-platelet ratio index (APRI) and the four-factor-based fibrosis index (FIB-4) were calculated. The outcome was the diagnosis of progressive liver fibrosis.

**Results:**

This study reviewed the data from 41 children with biliary atresia. APRI had 52% sensitivity and 83% specificity for progressive liver fibrosis, while FIB-4 had 83% sensitivity and 67% specificity. Their areas under the curve were not significantly different from those of conventional markers.

**Conclusion:**

Although they were not better than conventional markers, APRI and FIB-4 can be used as follow-up markers for progressive liver fibrosis in patients with biliary atresia, but their predictive value was moderate. Additional studies are necessary to determine whether they could be combined with other markers to improve their predictive value.

## Introduction

Biliary atresia (BA) is a rare liver disease in infants, leading to bile flow obstruction to the intestines and resulting in bile accumulation, liver cell injury, and liver fibrosis (1). Evaluating the extent of liver fibrosis in children with biliary BA is crucial for treatment and prognosis. Conventional histopathological assessment may be unsuitable for children with BA as it requires liver tissue biopsy (2). Therefore, finding non-invasive methods to predict the degree of liver fibrosis is very important in managing children with BA. Among the possible biomarkers, the aspartate aminotransferase (AST)-to-platelet (PLT) ratio index (APRI), previously studied in children with hepatitis B, hepatitis C, and BA, can serve as a non-invasive marker of fibrosis and cirrhosis (3). The four-factor-based fibrosis index (FIB-4) has been used to predict the degree of liver fibrosis in children with liver cystic fibrosis (4, 5). Therefore, we explored the predictive value of APRI, FIB-4, and serological markers for liver fibrosis in children with BA.

## Methods

This study retrospectively reviewed data from 41 children diagnosed with BA between March and December 2022. Liver tissue pathology specimens were obtained during surgery. This work has been carried out in accordance with the Declaration of Helsinki (2000) of the World Medical Association. This study was approved by the Ethics Committee of our hospital, and all participants provided written informed consent.

The serum markers measured within 1 week before the Kasai procedure or liver transplantation were collected: alanine aminotransferase (ALT), AST, γ-glutaryl transferase (GGT), total bilirubin (TBIL), direct bilirubin (DBIL), and PLT.

The pathological examinations were performed by a single pathologist with 20 years of experience, who assessed the liver fibrosis levels and inflammation grade based on the New Inuyama classification (6) using liver tissue pathology slides collected in this study. Fibrosis was staged as F0 (no fibrosis), F1 (fibrous portal expansion), F2 (bridging fibrosis, either portal-portal or portal-central linkage), F3 (bridging fibrosis with lobular distortion or disorganization), and F4 (cirrhosis). Inflammation was graded as A0 (no necro-inflammatory reaction), A1 (mild necro-inflammatory reaction), A2 (moderate necro-inflammatory reaction), and A3 (severe necro-inflammatory reaction). The patients were classified into non-progressive liver fibrosis (<F3) and progressive liver fibrosis (≥F3).

The APRI was calculated as APRI = (AST/upper limit of normal AST) × 100/PLT (7). FIB-4 was calculated as FIB-4 = (years of age × AST)/(PLT × √ALT) (8).

Statistical analysis was conducted using GraphPad Prism version 9.0.0 for Windows (GraphPad Software, San Diego, CA, USA) and MedCalc version 20.010 (MedCalc Software Ltd., Ostend, Belgium). The continuous variables were presented as means ± standard deviations or medians (interquartile ranges) and analyzed using the independent sample *t*-test or Mann–Whitney *U*-test. Categorical data were analyzed using the chi-squared test. Spearman correlation analysis was performed to examine the correlations between pairs of variables. Received operating characteristics (ROC) analysis was used to explore the predictive value of APRI, FIB-4, and serological markers for liver fibrosis levels. The Delong test was used to compare the area under the curve (AUC) of the biomarkers. Two-sided *P*-values <0.05 were considered statistically significant.

## Results

**Figure 1:**
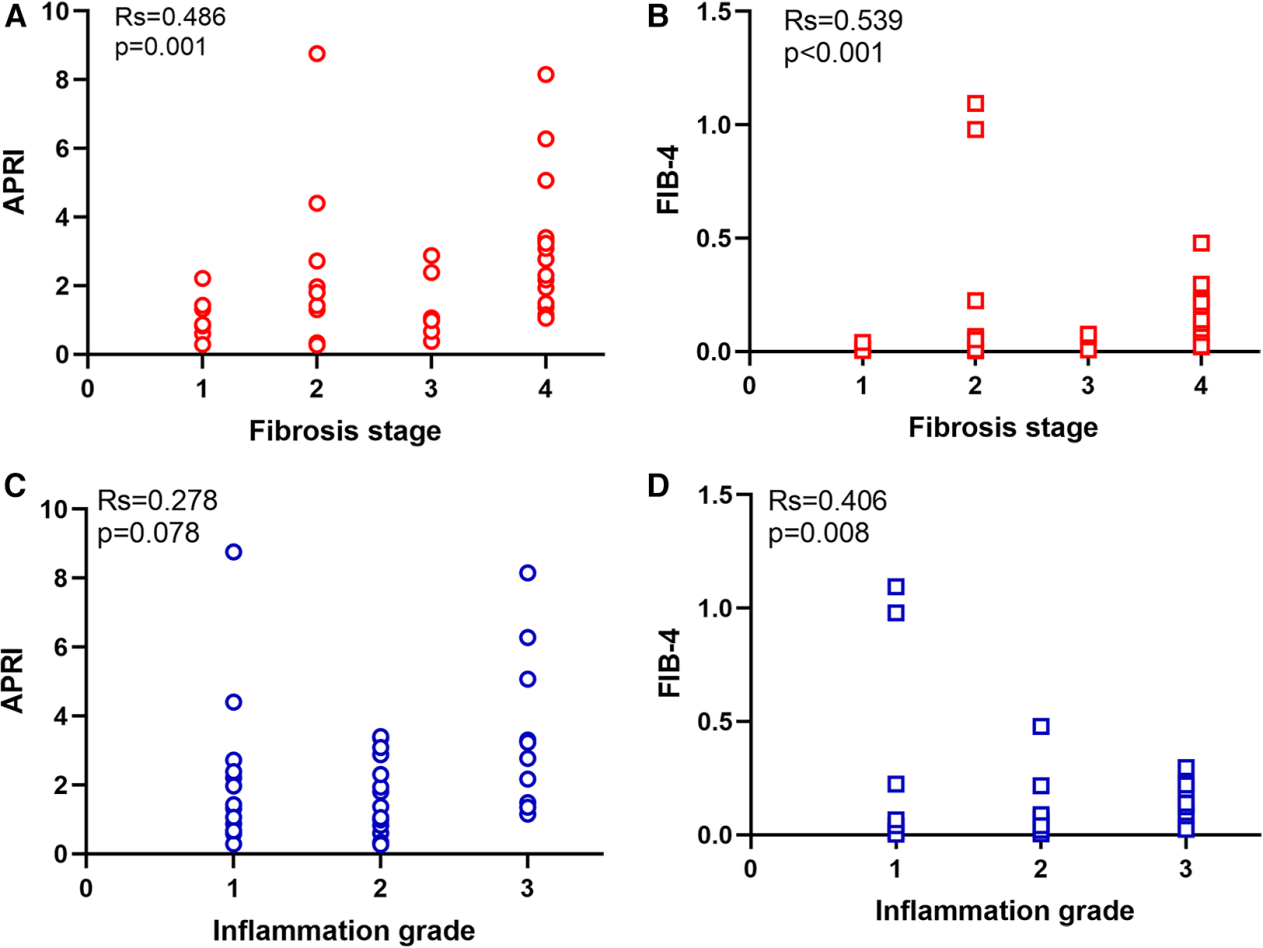
Distribution of APRI and FIB-4 in different fibrosis stage and inflammation grade. (**A**) APRI in different fibrosis stage. (**B**) FIB-4 in different fibrosis stage. (**C**) APRI in different inflammation grade. (**D**) FIB-4 in different inflammation grade.

The study included 23 patients with BA and progressive liver fibrosis and 18 with BA and non-progressive liver fibrosis. The between-group comparison is presented in Table 1. There were no significant differences between the two groups regarding age (at admission and surgery), sex, ALT, AST, GGT, TBIL, and DBIL (all *P* > 0.055). Compared with the non-progressive liver fibrosis group, the progressive liver fibrosis group showed lower PLT (255.0 ± 28.6 vs. 399.7 ± 59.8 × 10^9^/L, *P* = 0.039), higher APRI score (median, 2.3 vs. 11.32, *P* = 0.033), and higher FIB-4 score (median, 0.0805 vs. 0.0071, *P* = 0.019). Figure 1 shows the APRI and FIB-4 scores according to the fibrosis stage (Figures 1A,B) and inflammation grade (Figures 1C,D).

**Table 1:** Demographics and laboratory characteristics among BA patients.

	Total BA patients	Progressive liver fibrosis group (F3–4)	Non-progressive liver fibrosis group (F1–2)	*P*-value
*n*	41	23	18	
Sex (*n*)				0.194
Male	18	8	10	
Female	23	15	8	
Age at admission (days)	204.0 (48.0, 421.5)	268.0 (155.0, 487.0)	56.0 (45.25, 281.5)	0.072
Age at surgery (days)	206.0 (50.0, 423.5)	270.0 (157.0, 489.0)	58.0 (47.25, 283.5)	0.074
ALT (IU/L)	108.7 ± 10.2	108.1 ± 13.3	109.6 ± 16.3	0.943
AST (IU/L)	191.4 ± 15.5	207.8 ± 19.6	170.5 ± 24.6	0.237
GGT (IU/L)	388.0 (151.0, 698.5)	389.0 (232.0, 757.0)	368.5 (132.0,511.3)	0.344
TBIL (mmol/L)	170.7 (116.1, 240.8)	179.5 (89.4, 250.2)	158.1 (120.2,198.3)	0.674
DBIL (mmol/L)	126.7 (81.2, 208.4)	134.0 (79.3, 224.1)	110.8 (83.9,145.0)	0.478
PLT (×10^9^/L)	318.5 ± 32.4	255.0 ± 28.6	399.7 ± 59.8	0.039*
APRI	1.5 (1.0, 3.0)	2.3 (1.2, 3.3)	1.32 (0.6, 2.0)	0.033*
FIB-4	0.0402 (0.0051, 0.1337)	0.0805 (0.0203, 0.2021)	0.0071 (0.0030, 0.0557)	0.019*

BA, biliary atresia; APRI, aspartate aminotransferase to platelet ratio index; FIB-4, four-factor-based fibrosis index; ALT, alanine aminotransferase; AST, aspartate aminotransferase; GGT, *γ*-glutaryl transferase; TBIL, total bilirubin; DBIL, direct bilirubin; PLT, platelets.

^*^ *P*-value is significant at the 0.05 level (2-tailed).

Spearman correlation analysis showed that the APRI was significantly correlated to the fibrosis stage (*r *= 0.486, *P* = 0.001), ALT (*r* = 0.376, *P* = 0.015), AST (*r* = 0.512, *P* = 0.001), and PLT (*r* = 0.735, *P* < 0.001). FIB-4 was significantly correlated to the fibrosis stage (*r* = 0.539, *P* < 0.001), inflammation grade (*r* = 0.406, *P* = 0.008), GGT (−0.387, *P* = 0.012), and PLT (*r* = −0.828, *P* < 0.001). The APRI and FIB-4 were correlated (*r* = 0.818, *P* < 0.001) (Table 2).

**Table 2:** Spearman correlation analysis.

		Fibrosis stage	Inflammation grade	ALT	AST	GGT	TBIL	DBIL	PLT	APRI	FIB-4
Fibrosis stage	Spearman	1.000	0.748**	0.065	0.279	0.131	0.186	0.200	0.392*	0.486**	0.539**
	P	---	<0.001	0.686	0.078	0.414	0.245	0.210	0.011	0.001	<0.001
	n	41	41	41	41	41	41	41	41	41	41
Inflammation grade	Spearman	0.748	1.000	0.072	0.177	0.137	0.236	0.252	−0.230	0.278	0.406**
	P	<0.001	---	0.657	0.267	0.392	0.138	0.111	0.147	0.078	0.008
	n	41	41	41	41	41	41	41	41	41	41
ALT	Spearman	0.065	0.072	1.000	0.807**	0.281	0.345*	0.284	0.162	0.376*	0.87
	P	0.686	0.657	---	<0.001	0.075	0.0277	0.072	0.311	0.015	0.587
	n	41	41	41	41	41	41	41	41	41	41
AST	Spearman	0.279	0.177	0.807**	1.000	0.247	0.586**	0.536**	0.131	0.512**	0.205
	P	0.078	0.267	<0.001	---	0.120	<0.001	<0.001	0.413	0.001	0.199
	n	41	41	41	41	41	41	41	41	41	41
GGT	Spearman	0.131	0.137	0.281	0.247	1.000	0.235	0.223	0.413**	−0.206	−0.387*
	P	0.414	0.392	0.075	0.120	---	0.139	0.161	0.007	0.196	0.012
	n	41	41	41	41	41	41	41	41	41	41
TBIL	Spearman	0.186	0.236	0.345*	0.586**	0.235	1.000	0.972**	0.208	0.186	0.047
	P	0.245	0.138	0.027	<0.001	0.139	---	<0.001	0.192	0.245	0.772
	n	41	41	41	41	41	41	41	41	41	41
DBIL	Spearman	0.200	0.252	0.284	0.536**	0.223	0.972**	1.000	0.159	0.192	0.097
	P	0.210	0.111	0.072	<0.001	0.161	<0.001	---	0.321	0.229	0.546
	n	41	41	41	41	41	41	41	41	41	41
PLT	Spearman	−0.392*	−0.230	0.162	0.413**	0.413**	0.208	0.159	1.000	−0.735**	−0.828**
	P	0.011	0.147	0.311	0.007	0.007	0.192	0.321	---	<0.001	<0.001
	n	41	41	41	41	41	41	41	41	41	41
APRI	Spearman	0.486**	0.278	0.376*	−0.206	−0.206	0.186	0.192	−0.735**	1.000	−0.818**
	P	0.001	0.078	0.015	0.196	0.196	0.245	0.229	<0.001	---	<0.001
	n	41	41	41	41	41	41	41	41	41	41
FIB-4	Spearman	0.539**	0.406**	0.087	−0.387*	0.387*	0.047	0.097	−0.828**	0.818**	1.000
	P	<0.001	0.008	0.587	0.012	0.012	0.772	0.546	<0.001	<0.001	---
	n	41	41	41	41	41	41	41	41	41	41

APRI, aspartate aminotransferase to platelet ratio index; FIB-4, four-factor-based fibrosis index; ALT, alanine aminotransferase; AST, aspartate aminotransferase; GGT, *γ*-glutaryl transferase; TBIL, total bilirubin; DBIL, direct bilirubin; PLT, platelets.

* *P *< 0.05.

** *P* < 0.01.

**Figure 2:**
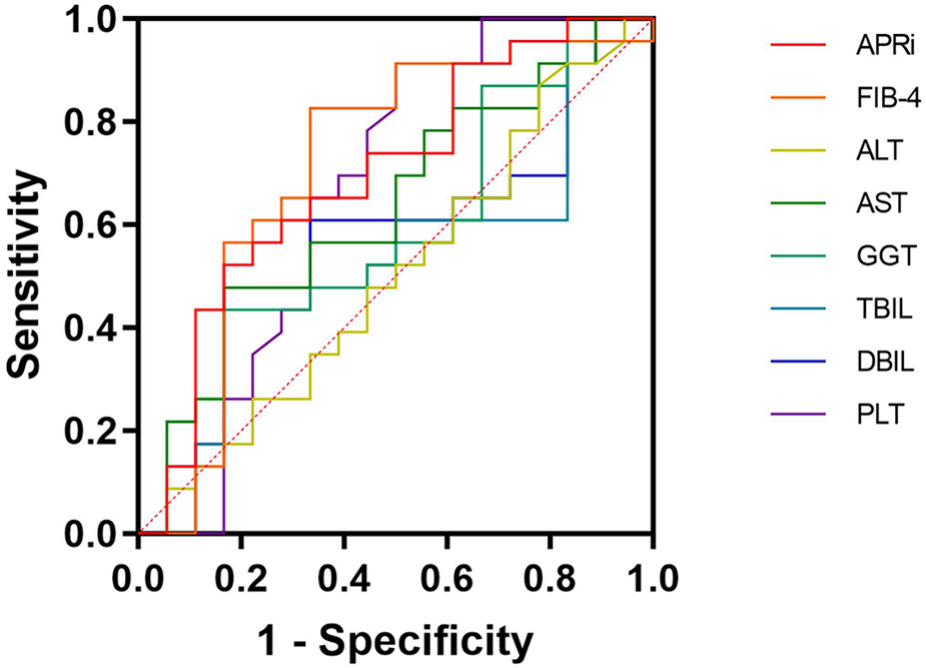
Receiver operating characteristics curve. APRI, aspartate aminotransferase to platelet ratio index; FIB-4, four-factor-based fibrosis index; ALT, alanine aminotransferase; AST, aspartate aminotransferase; GGT, *γ*-glutaryl transferase; TBIL, total bilirubin; DBIL, direct bilirubin; PLT, platelets.

The comparison of the AUCs between APRI and ALT, AST, GGT, TBIL, DBIL, and PLT showed no statistically significant differences (*P* = 0.11, *P* = 0.53, *P* = 0.40, *P* = 0.21, *P* = 0.28, and *P* = 0.32, respectively). Similarly, the comparison of AUC between FIB-4 and ALT, AST, GGT, TBIL, DBIL, and PLT also revealed no statistically significant difference (*P* = 0.07, *P* = 0.53, *P* = 0.44, *P* = 0.21, *P* = 0.30, and *P* = 0.65, respectively). The comparison of AUC between FIB-4 and APRI was not statistically significant (*P* = 0.74). APRI had 52% sensitivity and 83% specificity for progressive liver fibrosis, while FIB-4 had 83% sensitivity and 67% specificity (Figure 2 and Table 3).

**Table 3:** ROC analysis.

	AUC	95% CI	Cutoff	Sensitivity (%)	Specificity (%)	PPV (%)	NPV (%)
APRI	0.70	0.53–0.86	2.26	52.17	83.33	80.00	57.69
FIB-4	0.72	0.54–0.89	0.02	82.61	66.67	76.00	75.00
ALT	0.50	0.32–0.68	45.00	86.96	22.22	58.83	57.14
AST	0.63	0.46–0.81	229.50	47.83	83.33	78.57	55.55
GGT	0.59	0.41–0.77	537.00	43.48	83.33	76.92	53.57
TBIL	0.54	0.36–0.72	199.80	47.83	83.33	78.57	55.55
DBIL	0.57	0.38–0.75	164.20	47.83	83.33	78.57	55.55
PLT	0.66	0.48–0.85	397.00	91.3	50.00	70.00	81.81

AUC, area under the curve; CI, confidence interval; PPV, positive predictive value; NPV, negative predictive value; APRI, aspartate aminotransferase to platelet ratio index; FIB-4, four-factor-based fibrosis index; ALT, alanine aminotransferase; AST, aspartate aminotransferase; GGT, γ-glutaryl transferase; TBIL, total bilirubin; DBIL, direct bilirubin; PLT, platelets.

## Discussion

Patients with BA usually undergo the Kasai procedure to buy time pending liver transplantation. Patients with non-progressive liver fibrosis can undergo transplantation at an older age, but patients with progressive liver fibrosis must be carefully monitored to determine the optimal timing of transplantation. Even though conventional routine blood tests and liver function indicators (e.g., AST, ALT, GGT, TBIL, and DBIL) are useful in the diagnosis and monitoring of BA, these biochemical markers have limited diagnostic efficacy in identifying progressive liver fibrosis (F ≥ 3) and are unable to provide effective clinical assistance for accurate judgment in patients with BA (9).

Histopathological examination is the only definitive diagnostic tool for liver fibrosis, but the invasiveness of liver biopsy limits its use in neonates, and it cannot be repeated periodically in the context of long-term condition monitoring (2). Liver elastography assessed by ultrasound can provide a fair assessment of liver fibrosis (10, 11), including in children (12), but it is operator-dependent and has limited reliability and reproducibility (13). Still, specific ultrasound modalities have advantages and disadvantages. Vibration-controlled transient elastography can provide a quick bedside assessment but does not provide real-time ultrasound guidance and performs poorly in congestion, obesity, and inflammation (2). Two-dimensional shear-wave elastography can be implemented in regular scanners, shows the liver parenchyma, and can measure several levels at the same time, but it is affected by steatosis, inflammation, age, and BMI, and different manufacturers use different cutoff points (2). Point shear-wave elastography has the same advantages and disadvantages as two-dimensional shear-wave elastography, and it can evaluate a single region of interest (2). Shear-wave elastography combined with serum markers could be used to assess fibrosis after the Kasai procedure (14). Acoustic radiation force pulse imaging (ARFI) appears promising to evaluate liver fibrosis (15), but little data are available for its use in BA. Magnetic resonance elastography and diffusion-weighted imaging are promising modalities but are limited by scanner availability, high costs, long scanning times, and the necessity for holding breath, which is impossible in newborns and difficult in infants (2, 16, 17). As reviewed by Nallagangula et al. (18), Lew-Tusk et al. (19), and Ozdogan et al. (2), blood biomarkers for liver fibrosis include indirect markers (albumin, bilirubin, AST, ALT, GGT, ALP, and prothrombin time), direct markers (collagens, glycoproteins and polysaccharides, collagenases, hyaluronic acid, type IV collagen, procollagen III aminopeptide, laminin, YKL-40, MCP-1, sFas, CK18, and autotaxin), and combinational markers (APRI, AST/ALT, Bonacini index, ELF index, FIB-4, Fibro index, fibrometer test, FibroSpect II, Foma test, Hepascore, Fibrotest, and Lok index). Their AUCs are highly variable, even for a given biomarker (2, 18). Still, MMP-7 appears promising (20–27) and could be included in test packages or composite scores for BA, including in newborn screening (28). In addition, some of these tests are not routinely performed in the clinical setting or require special devices. Hence, the main advantage of the APRI and FIB-4 is that they can be calculated using routine blood test results without additional tests. It is an important point to consider in infants with an already highly morbid condition like BA. Of note, the present study did not evaluate the APRI and FIB-4 for diagnosing BA but for progressive liver fibrosis. Therefore, they could be of value for determining the timing of interventions like liver transplantation. Nevertheless, a major issue is that the available studies (including the present one) examined only a few modalities or biomarkers at a time; future studies should examine multiple imaging modalities, serum markers, and composite scores within the same patient samples and perform comparisons among them.

In the present study, APRI and FIB-4 showed a similar diagnostic value for progressive liver fibrosis (F ≥ 3; pathological diagnosis) compared with the traditional indicators, with FIB-4 outperforming APRI. Still, their AUCs were <0.75, indicating a suboptimal diagnostic value. These results are supported by a meta-analysis that revealed pooled sensitivity and specificity of 61% and 80% for significant liver fibrosis after surgery for BA (29). A study that used liver ultrasound for fibrosis diagnosis revealed AUCs of 0.897 for APRI and 0.856 for FIB-4 (14). However, it is worth noting that the predictive value of FIB-4 could be affected by age, as observed in adults (30). Since the patients in this study were relatively young, it could lead to lower FIB-4 values. In the previous studies, APRI and FIB-4 were evaluated postoperatively, while the present study evaluated these indexes before surgery. It will be necessary to examine such models in large-scale multicenter studies. Nevertheless, in the meantime, the APRI and FIB-4 can still be considered auxiliary monitoring biomarkers of liver fibrosis in children with BA, together with other indicators to reflect the situation of liver fibrosis in BA.

This study had limitations. It was a single-center study, and we enrolled all eligible patients during the study period. However, biliary atresia is a rare disease with an incidence of about 1 in 10,000, and it is not easy to collect cases. In addition, some patients had either passed away or returned to their residential areas during this time, making their inclusion unfeasible. Consequently, this situation introduced a bias as these patients could no longer be included in the consecutive enrollment process. These resulted in a small sample size. Even though enrollment was prospective, the available data were limited to the routine tests performed in infants with BA for ethical considerations against additional testing in infants with severe conditions and poor functional reserves. The study did not enroll control infants.

Since children with BA undergo the Kasai procedure and are observed until liver transplantation, and although they were not better than conventional markers, APRI and FIB-4 can be used as follow-up markers for progressive liver fibrosis in patients with biliary atresia, but their predictive value was moderate. Additional studies are necessary to determine whether they could be combined with other markers to improve their predictive value.

## Data Availability

The original contributions presented in the study are included in the article/Supplementary Material, further inquiries can be directed to the corresponding author.
